# Virulence, phenotype and genotype characteristics of invasive group B Streptococcus isolates obtained from Swedish pregnant women and neonates

**DOI:** 10.1186/s12941-022-00534-2

**Published:** 2022-10-13

**Authors:** Emily M. Huebner, Margrét Johansson Gudjónsdóttir, Matthew B. Dacanay, Shayla Nguyen, Alyssa Brokaw, Kavita Sharma, Anders Elfvin, Elisabet Hentz, Ysabella Raceli Rivera, Nicole Burd, Megana Shivakumar, Brahm Coler, Miranda Li, Amanda Li, Jeff Munson, Austyn Orvis, Michelle Coleman, Bo Jacobsson, Lakshmi Rajagopal, Kristina M. Adams Waldorf

**Affiliations:** 1 School of Medicine, University of Washington, Seattle, WA USA; 2 Department of Obstetrics and Gynecology, University of Washington, Seattle, WA USA; 3 Department of Pediatrics, Institute of Clinical Sciences, Sahlgrenska Academy, University of Gothenburg, Gothenburg, Sweden; 4 Department of Pediatrics, Region Västra Götaland, Sahlgrenska University Hospital, Gothenburg, Sweden; 5 Center for Global Infectious Disease Research, Seattle Children’s Research Institute, Seattle, WA USA; 6 Elson S. Floyd College of Medicine, Washington State University, Spokane, WA USA; 7 Columbia University, New York, NY USA; 8 Department of Psychiatry and Behavior Sciences, University of Washington, Seattle, WA USA; 9 Department of Pediatrics, University of Washington, Seattle, WA USA; 10 Department of Obstetrics and Gynecology, Institute of Clinical Sciences, Sahlgrenska Academy, University of Gothenburg, Gothenburg, Sweden; 11 Sahlgrenska Academy, University of Gothenburg, Gothenburg, Sweden

**Keywords:** *Streptococcus agalactiae*, Group B Streptococcus, Pregnancy, Neonate, Early-onset disease, Late-onset disease, Preterm labor, Preterm birth

## Abstract

Group B streptococci (GBS) are bacteria that can cause preterm birth and invasive neonatal disease. Heterogeneous expression of virulence factors enables GBS to exist as both commensal bacteria and to become highly invasive. A molecular epidemiological study comparing GBS bacterial traits, genotype and host characteristics may indicate whether it is possible to predict the risk of perinatal invasive GBS disease and more accurately target intrapartum antibiotic prophylaxis. A total of 229 invasive GBS isolates from Swedish pregnant women or neonates were assessed for virulence and phenotypic traits: hemolysis zone, hemolytic pigment (Granada agar), Streptococcus B Carrot Broth (SBCB) assay, CAMP factor, and hyaluronidase activity. Genes regulating hemolytic pigment synthesis (*covR/covS*, *abx1*, *stk1*, *stp1*) were sequenced. Of the virulence factors and phenotypes assessed, a Granada pigment or SBCB score ≥ 2 captured more than 90% of EOD isolates with excellent inter-rater reliability. High enzyme activity of hyaluronidase was observed in 16% (36/229) of the invasive GBS isolates and notably, in one case of stillbirth. Hyaluronidase activity was also significantly higher in GBS isolates obtained from pregnant/postpartum individuals versus the stillbirth or neonatal invasive isolates (p < 0.001). Sequencing analysis found that *abx1* (g.T106I), *stk1* (g.T211N), *stp1* (g.K469R) and *covS* (g.V343M) variants were present significantly more often in the higher (Granada pigment score ≥ 2) versus lower pigmented isolates (p < 0.001, each variant). Among the 203 higher Granada pigment scoring isolates, 22 (10.8%) isolates had 3 of the four sequence variants and 10 (4.9%) had 2 of the four sequence variants. Although heterogeneity in GBS virulence factor expression was observed, the vast majority were more highly pigmented and contained several common sequence variants in genes regulating pigment synthesis. High activity of hyaluronidase may increase risk for stillbirth and invasive disease in pregnant or postpartum individuals. Our findings suggest that testing for GBS pigmentation and hyaluronidase may, albeit imperfectly, identify pregnant people at risk for invasive disease and represent a step towards a personalized medical approach for the administration of intrapartum antibiotic prophylaxis.

## Background

*Streptococcus agalactiae*, or group B streptococcus (GBS), colonizes the lower genital tract of approximately 18% of pregnant women [1]. However, GBS can become highly pathogenic if it ascends into the uterus and infects the placenta and fetus. Adverse maternal and neonatal outcomes such as maternal sepsis, preterm birth, stillbirth, and invasive early-onset disease (EOD) of the neonate are linked to invasive or ascending GBS infections [2, 3]. EOD presents in the first week of life as sepsis that may be complicated by pneumonia or meningitis and is thought to arise from a GBS infection in utero or during birth. EOD can be reduced by administration of intrapartum antibiotic prophylaxis (IAP) to pregnant women harboring GBS in the lower genital tract or who have EOD risk factors (e.g., prolonged rupture of membranes) [4, 5]. Unfortunately, IAP does not impact late-onset disease (LOD), which presents between 7–89 days of life and is thought to be acquired by the neonate after birth; further, IAP is administered too late to prevent GBS-associated preterm birth and stillbirth [6, 7]. Globally, the guidelines for IAP administration differ and can be based on the results of rectovaginal GBS cultures performed universally on all pregnant individuals, as in the U.S.; alternatively, IAP may be administered based on the development of a set of clinical risk factors for EOD, as in Sweden. A better understanding of GBS virulence factors and their role in perinatal invasive GBS disease may improve the identification of pregnancies at high-risk for GBS invasive disease to better target IAP and direct vaccine development. Historically, the main GBS virulence factor associated with adverse maternal-neonatal outcomes was the GBS capsular polysaccharide (CPS) serotype. GBS are currently divided into ten serotypes (Ia, Ib, II-IX) based on CPS composition. Most neonatal infections are caused by serotypes Ia, Ib, II, III, and V. Serotype III is particularly pathogenic and has predominated in LOD and cases of neonatal meningitis [8–12]. Protein-conjugated CPS vaccines are under development including a hexavalent CRM197-CPS (Ia, Ib, II – V) [13, 14]. A maternal GBS vaccination strategy based on CPS prevalent today may result in selection pressure that promotes serotype emergence for those not targeted by the vaccine.

A key GBS factor associated with virulence is the β-hemolysin/cytolysin (hereafter referred to as the hemolysin), whose expression is conserved in many clinical isolates. Hemolysin was discovered to be a surface-associated ornithine rhamnolipid pigment, which is cytotoxic to red blood cells and many other host cells, promoting bacterial dissemination [15–17]. Hemolysin expression is regulated by the two-component system known as covR/S [18, 19]. This system represses expression of the cyl operon encoding enzymes and factors necessary for hemolysin biosynthesis [15, 18–20]. In many hyperhemolytic and hyperpigmented clinical isolates, single nucleotide polymorphisms in *covR* or *covS* (leading to altered amino acid sequences) or in the promoter region were identified and attributed to these phenotypes [15, 21, 22]. Other genes, including *abx1* and *stk1*/*stp1* also regulate hemolysin production through their effects on covR expression [23, 24]. Many studies have identified hyperpigmented and hyperhemolytic isolates from patients with GBS disease, women with preterm labor or neonates with invasive GBS disease [15, 21]. Hyperhemolytic GBS strains have also been isolated from ill nonpregnant adults, with fatality reported in one case [22, 25, 26]. Nonhemolytic isolates have been described as less frequently associated with invasive disease, though a few pathogenic strains have been reported [27, 28]. For example, a novel serotype V clinical GBS strain (GB37) isolated from a neonate with EOD is nonhemolytic [27]. This phenotype was attributed to a single nucleotide polymorphism in covS. Intriguingly, this isolate expressed exceptionally high GBS hyaluronidase enzyme activity (also called HylB). HylB directs immune evasion through its ability to degrade host hyaluronan into immunosuppressive disaccharides that block toll-like receptor (TLR) 2− and 4− mediated immune responses [29]. Although one study identified hylB in only 12 of 154 isolates from patients in a Kuwait maternity hospital [30], another study by Vornhagen et al. reported significantly higher hyaluronidase activity in clinical isolates from cases of invasive GBS disease compared to asymptomatically colonizing isolates [31]. When further stratified by clinical outcome, high hyaluronidase activity was also significantly increased among cases of preterm labor. These findings suggest that hyaluronidase may confer GBS with a virulence profile that elicits a different host response when compared to those observed during infection with hyperhemolytic strains.

Significant knowledge gaps exist regarding the coordination of GBS pathogenesis by many known virulence factors [20], including hemolysin [15, 17, 32] and hyaluronidase [27, 29, 31, 33] to direct perinatal invasive disease. Our understanding of these factors comes from studies using clinical isolates that may not appropriately capture the diversity of GBS in human populations. A molecular epidemiological study comparing GBS bacterial traits, genotype and host characteristics may indicate whether it is possible to predict risk of perinatal invasive GBS disease and more accurately target IAP. Our first study objective was to determine the degree of diversity in expression of GBS virulence factors, specifically hemolysin and hyaluronidase, using routine microbiological assays in a large biobank of invasive GBS isolates from pregnant individuals, neonates with EOD or LOD and stillborn fetuses. We also wanted to determine the discriminatory ability of routine microbiological assays, some which measure the same biological pathway (hemolysin production), to capture the greatest number of invasive isolates. Finally, we sought to correlate low- and high expression of the GBS hemolysin with common sequence variants in genes controlling the expression of CovR/CovS and clinical outcome. Association of GBS virulence factors, bacterial phenotype, genotype and clinical characteristics has not previously been done in a large GBS biobank.

## Materials and methods

### Study design

Invasive GBS isolates (N = 233) were collected from six microbiology laboratories serving 13 hospitals in Western (Västra Götaland) and Southwest (Halland) Sweden in 1988–2001 and 2004–2009, as previously described [8, 34, 35]. GBS isolates were collected from the blood, cerebrospinal fluid (CSF) and synovial fluid. Four neonates with LOD GBS disease had two isolates each; three neonates had isolates obtained from blood and CSF, and one neonate had isolates from synovial fluid and blood. All other isolates were obtained from a single site. Isolates were identified as GBS according to colony morphology, microscopy following Gram´s stain of smears, and co-agglutination with group-specific reagents (Streptex; Murex Biotech, Dartford, UK). The isolates were initially stored in a lyophilizing broth (nutrient broth [Difco], horse serum [Håtunalab], glucose, and sterile water) manufactured by the Department of Clinical Microbiology, Sahlgrenska University Hospital, Gothenburg, Sweden, before they were freeze-dried by Culture Collection University of Gothenburg. All isolates underwent GBS serotyping. Serotyping results were compared to a previously published cohort of commensal GBS isolates that were obtained from nearly all Swedish pregnant women and their infants that delivered in a single week in 2005 [36].

### Analysis of virulence factors

**Fig. 1:**
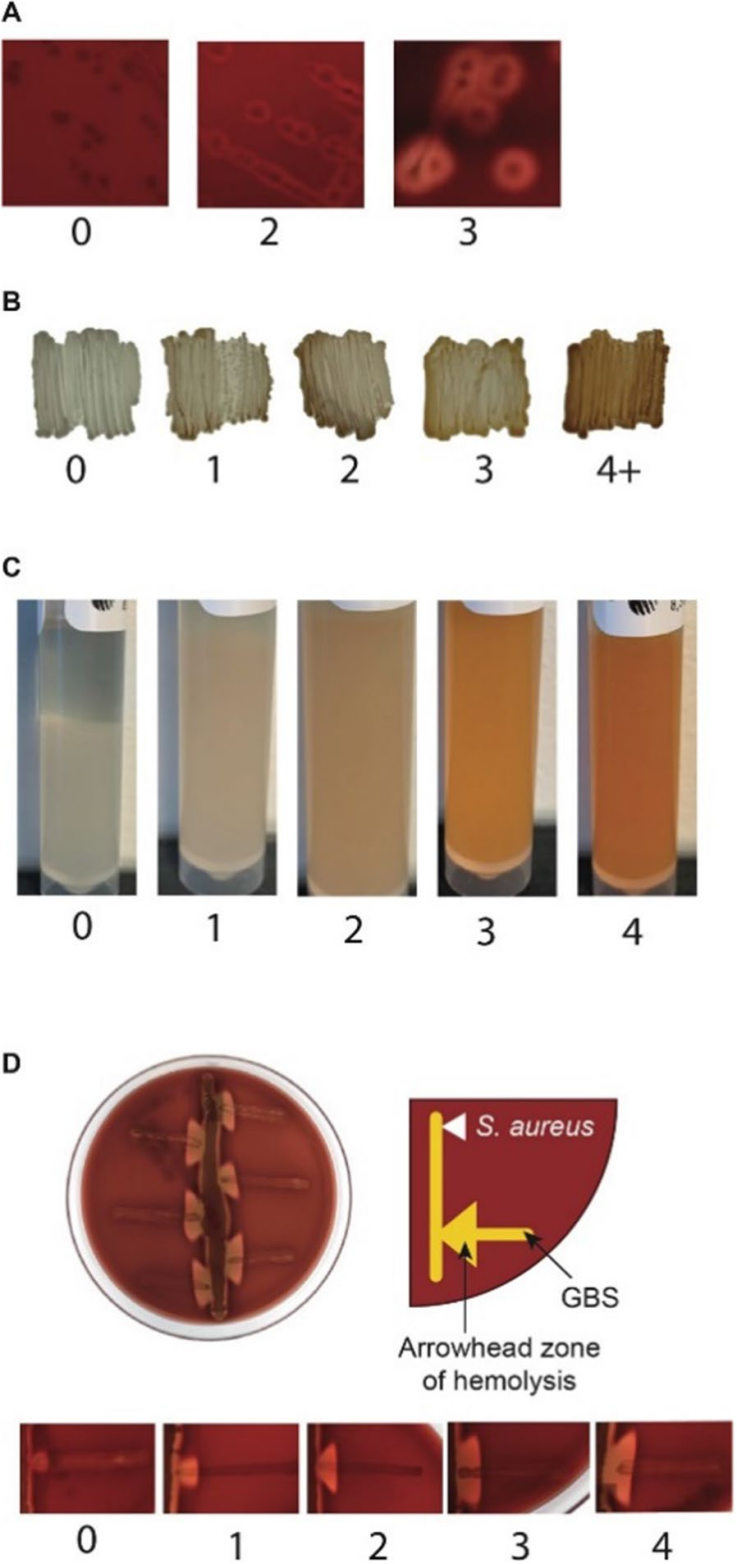
Scoring examples of Group B Streptococcus (GBS) β-hemolysis, Granada pigment, Strep B Carrot Broth (SBCB) assay and CAMP Factor. Examples of the range of scores are shown that were associated with β-hemolysis (**A**), pigment on Granada agar (**B**), orange pigment using the SBCB assay (**C**) and CAMP factor (**D**). In panel D, an illustration is shown of the vertical *Staphylococcus aureus (S. aureus)* streak near the horizontal GBS streak, which can produce the CAMP factor effect that appears as an arrowhead

**Fig. 2:**
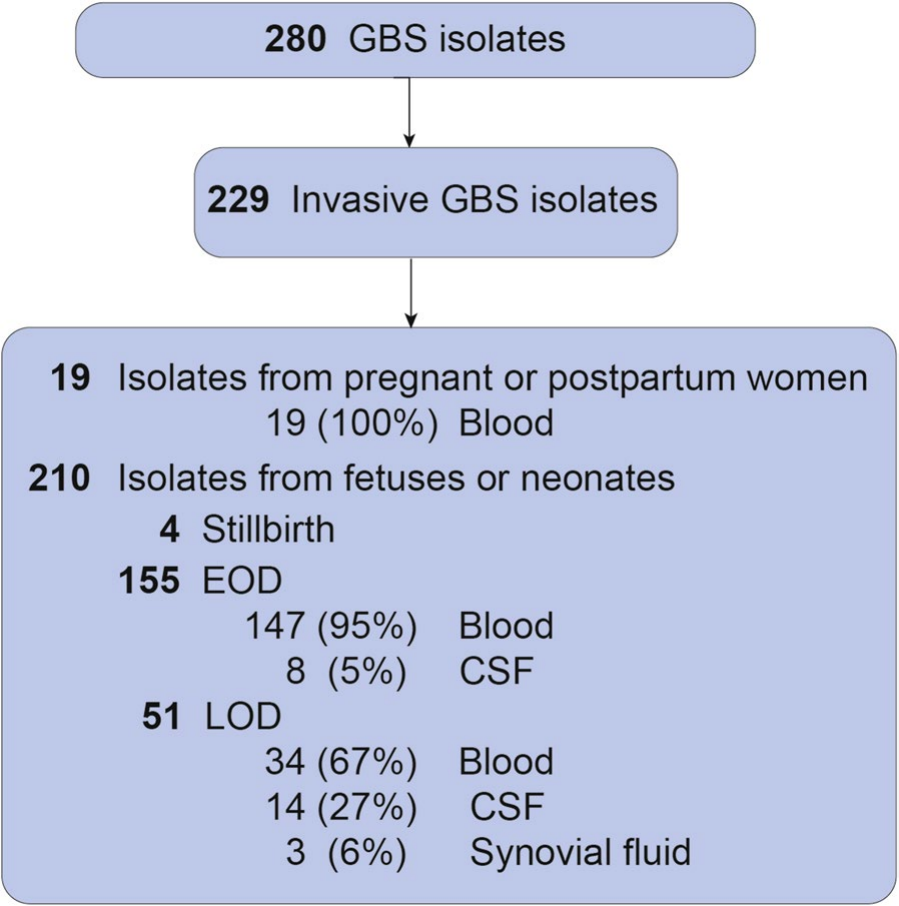
Study groups and sources of GBS isolates. The number of invasive GBS isolates and biological source of the isolate is shown for each clinical group

Invasive isolates were sent from Sweden to the University of Washington as freeze-dried samples. Isolates were reconstituted with tryptic soy broth (TSB; BD LLC, Franklin Lakes, US) and incubated at 37 °C overnight. Frozen stocks of each isolate were generated by diluting overnight cultures 1:1 with 50% glycerol in TSB and frozen at −80 °C. Beta-hemolysis was assessed by streaking isolates for single colonies individually on Sheep’s blood agar plates (Thermo Fisher Scientific Inc., Waltham, US) and scoring the zone of clearance appearing around each isolate (Fig. 1A). GBS contains a unique, red polyenic pigment called Granadaene, which is the hemolysin, and is particularly evident in certain media. Pigment production was determined via two separate methods: 1) by streaking isolates on Granada agar (Hardy Diagnostics, Santa Maria, U.S.; (Fig. 1B), and 2) by inoculating carrot broth (Strep B Carrot Broth™ One-Step (SBCB), Hardy Diagnostics, Santa Maria, U.S.) with a single GBS colony (Fig. 1C).

In parallel with these tests to evaluate pigment and hemolysis, we performed a test of Christie-Atkins-Munch-Peterson (CAMP) factor activity; although CAMP factor activity is not thought to be an essential virulence factor for GBS [37, 38], it does have membrane permeabilizing activity and has been a routine method for confirmation of GBS identity and a measure of hemolysis. To score CAMP factor activity, we plated each GBS isolate on Sheep’s blood agar with a 2 mm perpendicular streak of *Staphylococcus aureus* (Fig. 1D); GBS and *S. aureus* act synergistically to create a zone of enhanced hemolysis, which is the scored area [37]. All plates and media were incubated at 37 °C for 24 h.

Bacterial assay scoring was evaluated (0–4 + hemolysis, 0–4 + Granada pigment, 0–4 + SBCB pigment, and 0–4 + CAMP factor effect) by two individuals, who were blind to the group assignment. Reference strains with pre-determined grades were used for standardization: COH1 (hemolysis, 2 + ; CAMP factor, 3 + ; Granada pigmentation, 3 + ; SBCB pigmentation, 3 +) and NCTC 10/84 (hemolysis, 3 + , CAMP factor, 0; Granada pigmentation, 4 + ; SBCB pigmentation, 4 +). Examples of scores for each test are shown in Fig. 1. Although reference strains were used to establish baselines for scoring, occasional disagreements in grade occurred. Disagreements were discussed and adjudicated by a third grader.

### Hyaluronidase expression

Hyaluronidase assays were performed as previously described [31]. All chemicals were obtained from Sigma-Aldrich, Saint Louis, U.S. GBS strains were grown overnight in TSB and centrifuged to obtain culture supernatants. Commercial hyaluronidase (from bovine testes) was used to make standards at concentrations of 0, 0.5, 1, 2.5, and 5 mg/ml in TSB. Next, 200 µl of 1.25 mg hyaluronic acid (dissolved in 36.0 mg/ml monobasic sodium phosphate, pH 5.35) was added to each GBS culture and incubated at 37 °C for 45 min. Following incubation, 50 µl of 0.8 M sodium tetraborate (pH 9.1) pre-warmed to 95 °C was added to each sample and to hyaluronidase standards. Samples and standards were incubated at 95 °C for 5 min, following which 1.5 ml of 1.0% 4-dimethylaminobenzaldehyde (dissolved in 15.3 M glacial acetic acid and 1.25 M hydrochloric acid) was added. A color change to magenta indicated hyaluronidase activity. Immediately following the color change, absorbance at 585 nm was measured. A standard curve was created to extrapolate hyaluronidase concentration in each GBS culture supernatant [31]. The GBS clinical WT strain GB37 and isogenic hyaluronidase deficient GB37Δ*hylB* were used as positive and negative controls, respectively.

### Gene sequencing

The *covRS*, *abx1*, *stk1* and *stp1* genes were amplified for each of the invasive isolates using primers (Table 1) and NEB’s Q5 High Fidelity Mastermix.

**Table 1:** Primer sequences

Genes	Gene segment	Primer sequence
*covRS*	SAK_1640 promoter 5' end	5'-CGTATTGAGCGTTTGCGT-3'
	SAK_1640 promoter extended primer 1	5'GAGATGGCACGTGTTACTTACAG-3’
	*covr* Promoter 5' SphI	5'-GATCGCATGCTTAATAACATCAGTTGATAT-3'
	*covs* Downstream R	5'-GGACAACGCATGTCAACACCCC-3'
	*covrs* F Seq 3	5'-GATTCTGTTATGGATATTGTAGC-3'
	*covrs* F Seq 4	5'-GTTTTTATCTTATTTTTTAGCC-3'
	*covrs* F Seq 5	5'-GATTATGATGGAAGTTTTAGGG-3'
	*covrs* F Seq 6	5'-CATGATCTTAATTGATAACGC-3'
	BA *covS*	5’-GAGGCAATTCTTCCAAAC-3'
*abx1*	KO_abx1_5	5'-AGGCTGTTATTCATTAGGTCACTTG-3'
	KO_abx1_3	5'-AACCGTAAATACAAGAAACAGATGC-3'
	Above_abx1	5'-AACAGTAGTTCCGGCCATTAG-3'
	Mid_abx1	5'-TGGAGAATTAGGAACCTTTAGT-3'
*stk1/stp1*	*STK.STP1*-Forward	5'-ACCACCCCAATTTTGAGCAAG-3'
	*STK.STP1*-REVERSE	5'-CTAGGCATGGTCTCTGCCAT-3'

Primer sequences used to amplify genes for *covR, covS*, *abx1*, *stk1* and *stp1* are shown

Manufacturer’s recommendations were followed for the PCR reaction. PCR amplified samples were confirmed by gel electrophoresis and then column purified using a Genejet PCR purification kit (ThermoFisher Scientific, Waltham, MA, U.S.). After measuring the concentration of the purified samples by UV–Vis Spectroscopy, samples were prepared for Sanger Sequencing. Samples were diluted and mixed individually with primers that anneal to multiple overlapping locations (Table 1). Primer extension sequencing was performed by Genewiz, Inc (Seattle, WA) using Applied Biosystems BigDye version 3.1. Reactions were run on Applied Biosystem's 3730xl DNA Analyzer. Isolate gene sequences were obtained from Genewiz. FASTA files were read into Snapgene software and aligned with the genome sequence of *covR* and *covS f*rom GBS COH1 [39]. Chromatogram files were separately analyzed to confirm the sequences. Mutations were recorded. Synonymous mutations were identified separately from non-synonymous mutations and location noted. The mutation nucleotide positions are recorded in reference to the start of the forward primer used in the PCR reactions to generate the amplicon, which was upstream of the open reading frame (ORF). The codon position of mutations was calculated utilizing the mutation nucleotide position and the start codon nucleotide position.

### Statistical analysis

Estimation of the inter-rater reliability (IRR) for virulence/phenotypic scoring used a weighted Kappa provided by the “cohen.kappa” function from the “psych” R package. Given the ordinal nature of the data, we examined differences between neonatal and maternal isolates for virulence/phenotypic scores with a Kruskall-Wallis test. We also used a Fisher’s exact test to compare proportions of isolates with specific serotypes. A p-value less than 0.05 was considered significant.

### Ethics statement

The study was conducted in accordance with the Declaration of Helsinki and the research was approved on 01/25/2019 by the Swedish Ethical Review Authority (EPM 2019–00,549). Approval for the work at the University of Washington was obtained on 08/09/2021 from the University of Washington Human Subjects Division (STUDY00008540). At the Seattle Children’s Research Institute, approval for the work was obtained on 10/28/2019 from the Seattle Children’s Institutional Review Board (STUDY00002171). Isolates were approved for shipping by the U.S. Centers for Disease Control.

## Results

### Clinical characteristics and CPS serotypes

A total of 233 invasive GBS isolates were collected from neonates or pregnant/postpartum women (Table 2). In 4 subjects, two isolates were obtained from the same individual. Exclusion of 4 duplicate isolates for statistical analyses left 229 invasive GBS. Biological source and serotype distribution of the invasive isolates are shown (Fig. 2, Table 3).

**Table 2:** Clinical outcomes associated with the invasive GBS isolates

Outcome	EOD (N = 155)	LOD (N = 51)	Maternal (N = 19)
Gestational age at birth (weeks)	37.5 ± 4.1	36.9 ± 5.1	–
Preterm birth	45 (29)^a^	17 (33)	–
Sepsis	147 (94)	34 (66)	19 (100)
Meningitis	9 (6)	17 (33)	0 (0)
Pneumonia	7 (4)	2 (4)	0 (0)
Septic arthritis	0 (0)	3 (5)	0 (0)
Chorioamnionitis	–	–	9 (47)
Postpartum endometritis	–	–	10 (53)
Death	8 (5)	3 (5)	< 3 (< 16)

Clinical outcomes are shown for the EOD, LOD and maternal invasive isolates. Note that we have not shown the 4 stillbirths in this table to prevent identification of individual cases. The mean ± standard deviation is shown for the neonatal gestational age at birth. All other numbers reflect counts (percentage)

*EOD* Early-onset disease; *LOD* Late-onset disease

^a^One case of EOD had missing information on gestational age

**Table 3:** GBS serotype counts in Swedish invasive and commensal isolates

Sero-type	EOD (N = 155)	LOD (N = 51)	Maternal (N = 19)	Stillbirth (N = 4)	Total GBS Invasive Isolates (N = 229)	Swedish GBS Commensal Isolates (N = 356)	p-value, total invasive vs. commensal
Ia	30 (19.4%)	4 (7.8%)	1 (5.3%)	1 (25%)	36 (15.7%)	39 (11%)	0.1
Ib	8 (5.2%)	1 (2.0%)	5 (26.3%)		14 (6.1%)	46 (13%)	0.008
II	8 (5.2%)	1 (2.0%)	1 (5.3%)	1 (25%)	11 (4.8%)	57 (16%)	< 0.001
III	78 (50.3%)	36 (70.6%)	3 (15.8%)		117 (51.1%)	85 (24%)	< 0.001
IV	3 (1.9%)	2 (3.9%)	2 (10.5%)		7 (3.1%)	53 (15%)	< 0.001
V	25 (16.1%)	5 (9.8%)	7 (36.8%)	2 (50%)	39 (17.0%)	68 (19%)	0.58
VII	1 (0.6%)				1 (0.4%)	2 (0.5%)	1
VIII		1 (2.0%)			1 (0.4%)	4 (1%)	0.65
IX	1 (0.6%)	1 (2.0%)			2 (0.9%)	0 (0%)	0.15
NT	1 (0.6%)				1 (0.4%)	2 (0.8%)	

This table presents a comparison of serotypes between Swedish GBS invasive and commensal isolates. The percent of serotypes in Swedish commensal GBS isolates (collected in 2005) was previously published and is presented here for comparison with the Swedish invasive GBS isolates [36]. The N in each serotype group of the Swedish commensal GBS isolates was estimated from the reported percent. In addition, serotyping of a subset of the invasive GBS isolates was previously published [8]. Fisher’s exact test was performed to compare the proportion of serotypes within the invasive and commensal GBS isolates of each serotype group (Groups Ia, Ib, II, III, IV and V) with multiple hypothesis correction for 6 tests. Serotype VI was not observed in these isolates

*EOD* Early-onset disease; *LOD* Late-onset disease; *NR* Not reported. *NT* Non-typeable

One EOD GBS isolate from blood was non-typeable. Serotype III was the most frequent among typeable invasive isolates (117/228, 51%) and particularly prevalent in the LOD group (36/51, 70%). All 22 GBS isolates extracted from CSF were obtained from neonates; fourteen of these were serotype III (14/22; 64%) and 14 were associated with LOD GBS disease (14/22; 64%). All isolates from pregnant or postpartum individuals were obtained from maternal blood and serotype V occurred most frequently (7/19, 37%). We compared the distribution of serotypes for all invasive isolates to published data from a Swedish commensal GBS group collected within the same period (Table 3) [36]. There were significant differences in the distribution of serotypes II, III and IV across the invasive and historical commensal groups with a higher proportion of serotypes II and IV in the commensals and a higher proportion of serotype III in the invasive GBS isolates (all, p < 0.001).

### Virulence factor expression in the GBS invasive isolates

**Fig. 3:**
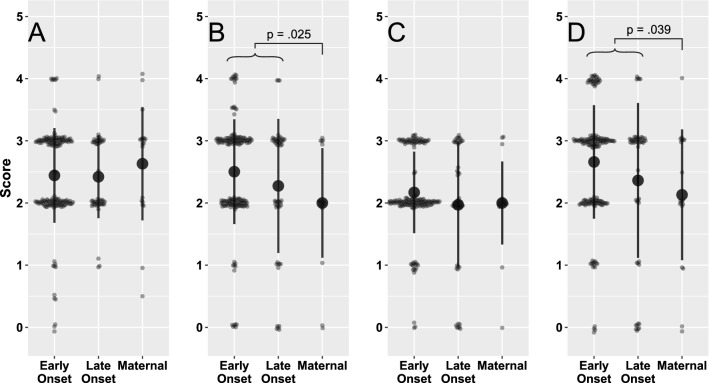
Virulence factor scoring of the GBS invasive isolates by test type. A dot plot of scores with mean and one standard deviation error baris shown for: **A** CAMP factor score on sheep blood agar, **B** Granada pigment score, **C** β-hemolysin activity on sheep blood agar, and **D** SBCB assay

We scored hemolysis, pigment production, CAMP factor, and SBCB for each GBS strain to determine if expression of key virulence factors correlated with GBS invasive disease (Fig. 1, Fig. 3). Using a weighted kappa, we found high levels of agreement across two independent raters (hemolysis = 0.94, Granada pigment = 0.96, CAMP factor = 0.97, SBCB = 0.91). Compared to the invasive maternal isolates from pregnant and postpartum individuals, there was a significantly higher Granada pigment (χ^2^ = 5.05, p = 0.03) and SBCB (χ^2^ = 4.25, p = 0.04) score in the neonatal invasive isolates; no significant differences between maternal and neonatal isolates were observed for hemolysis and CAMP factor scores. Of the phenotypic and virulence factors assessed, a Granada pigment score ≥ 2.0 captured the greatest proportion of invasive isolates: all invasive: 89% (203/229); EOD: 92% (143/155). An SBCB score of ≥ 2.0 captured 81% (186/229) of invasive isolates and 93% (144/155) of EOD isolates. No significant differences in virulence factor expression were found between EOD and LOD invasive isolates.

**Fig. 4:**
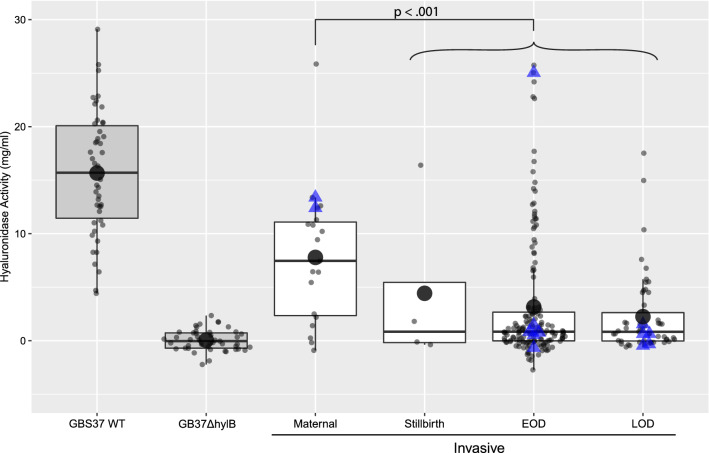
Hyaluronidase enzyme activity (boxplot: median, thick line; mean, large dot) is shown for each isolate by invasive GBS isolate group (maternal, stillbirth, EOD and LOD groups) in comparison to the GB37 wild-type (high hyaluronidase activity) and GB37ΔhylB (low hyaluronidase activity). The hyaluronidase enzyme activities of GB37 WT and GB37Δ*hylB* were assayed on each plate as a control. All GBS isolates with a pigment score greater than zero are shown as small dots. Non-pigmented GBS isolates are shown as blue triangles (Granada pigment score = 0). Note that 451 units of hyaluronidase activity/mg is equivalent to 1 mg/ml of hyaluronidase. We considered a hyaluronidase enzyme activity of > 10 mg/mL as “high”

GBS hyaluronidase enzyme activity reportedly promotes immune suppression during a GBS infection, thereby representing a novel virulence factor. A non-hemolytic GBS isolate, called GB37, was identified that overexpresses hyaluronidase [27, 29, 31, 33]. Whether other non-hemolytic GBS invasive isolates also overexpress hyaluronidase to enhance virulence is unknown. In our biobank of perinatal invasive GBS isolates, a high hyaluronidase activity (> 10 mg/mL) occurred in 15.7% (36/229) of all invasive isolates and 17.4% (4/23) of non-pigmented isolates (blue triangles, Fig. 4). This data indicates that high hyaluronidase activity can occur in GBS strains with and without pigment.

Interestingly, maternal isolates expressed significantly greater hyaluronidase enzyme activity than neonatal and fetal (stillbirth) isolates (χ^2^ = 12.53, p < 0.001, Fig. 4). However, in a single case of stillbirth, GBS hyaluronidase activity was very high in a pigmented strain (16.4 mg/mL). There were no differences in hyaluronidase enzyme activity between EOD and LOD invasive isolates. In summary, we found a continuum of hyaluronidase activity with high levels in both pigmented and non-pigmented GBS isolates suggesting that hyaluronidase is a commonly expressed virulence factor that is not always regulated by CovR/S.

**Fig. 5:**
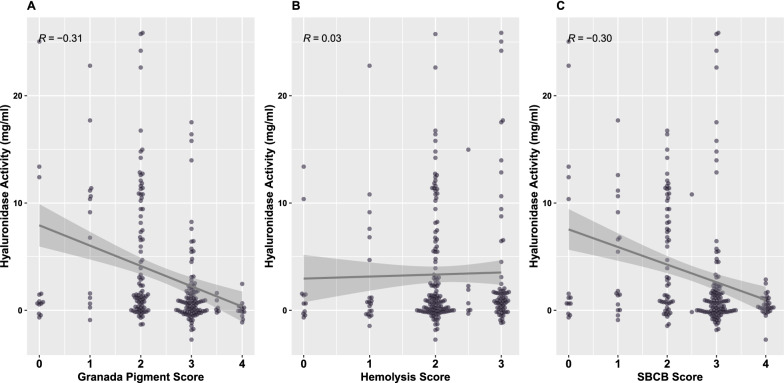
Correlation between hyaluronidase activity and Granada pigment, hemolysis, and SBCB scores

Next, we analyzed the correlation of virulence factor expression with hyaluronidase activity. There was a significant negative correlation between hyaluronidase activity and Granada pigment (r = −0.31, p < 0.001) and hyaluronidase activity and SBCB score (Fig. 5; r = −0.30, p < 0.001), but not between hyaluronidase activity and the hemolysis score. If we removed GBS isolates with hyaluronidase activity greater than 20 mg/ml (N = 6), the relationships remained significant (Granada: r = − 0.27, p < 0.001; SBCB: r = − 0.32, p < 0.001). Note the non-pigmented, non-hemolytic isolates that are indicated by a score of zero in Fig. 5, some of which have low hyaluronidase activity. The molecular basis for their invasive phenotype cannot be explained by either expression of the ß-hemolysin or hyaluronidase.

**Fig. 6:**
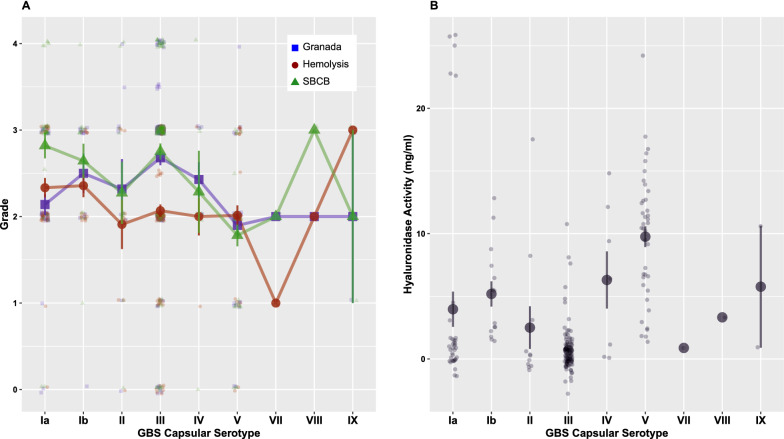
Assay grade scores (**A**) and hyaluronidase enzyme activity (**B**) are shown by GBS capsular serotype (Ia-IX). Serotype VI was not observed in these isolates. The symbols represent the mean assay grade (**A**) or hyaluronidase activity (**B**) with error bars indicating 1 standard error of the mean

Next, we analyzed the relationship between assay grades and hyaluronidase activity across GBS capsular serotypes. The mean and distribution of the graded scores for Granada pigment, hemolysis, SBCB, and hyaluronidase enzyme activity within each serotype is presented in Fig. 6. To reduce the complexity of analyses involving multiple serotypes, some of which were infrequent, we focused analysis on the two most frequent serotypes III and V and compared them to isolates in all other serotypes. In serotype III, Granada pigment scores were significantly higher and hyaluronidase activity significantly lower than in all other serotypes (Table 4). In serotype V, the mean hyaluronidase enzyme activity was significantly higher and Granada pigment and SBCB scores significantly lower than in all other serotypes (Table 4). In this cohort of invasive GBS isolates, serotypes III and V were notable for their contrasting Granada pigmentation and hyaluronidase activity.

**Table 4:** Comparison of Assay Grade and Hyaluronidase Activity by GBS Serotype

	Serotype III N = 117		Serotype V N = 39		All other Serotypes N = 72		Serotype IIIvs. Other		Serotype V vs. Other	
Variable	M	SD	M	SD	M	SD	t	p	t	p
Granada	2.68	0.93	1.90	0.75	2.26	0.81	3.28	0.001	− 2.34	0.022
Hemolysis	2.07	0.78	2.01	0.73	2.24	0.70	−1.53	0.127	− 1.56	0.123
SBCB	2.75	1.01	1.78	0.78	2.62	0.95	0.92	0.360	− 4.96	< 0.001
Hyaluronidase Activity (mg/ml)	0.71	1.79	9.76	5.15	4.21	6.86	−4.24	< 0.001	4.81	< 0.001

This table presents an analysis of the hyaluronidase activity, Granada pigment and SBCB scores from GBS isolate serotype III and serotype V groups compared to all other isolates

### Sequence variants in the covR, covS, abx1, stk1 and stp1 genes

**Fig. 7:**
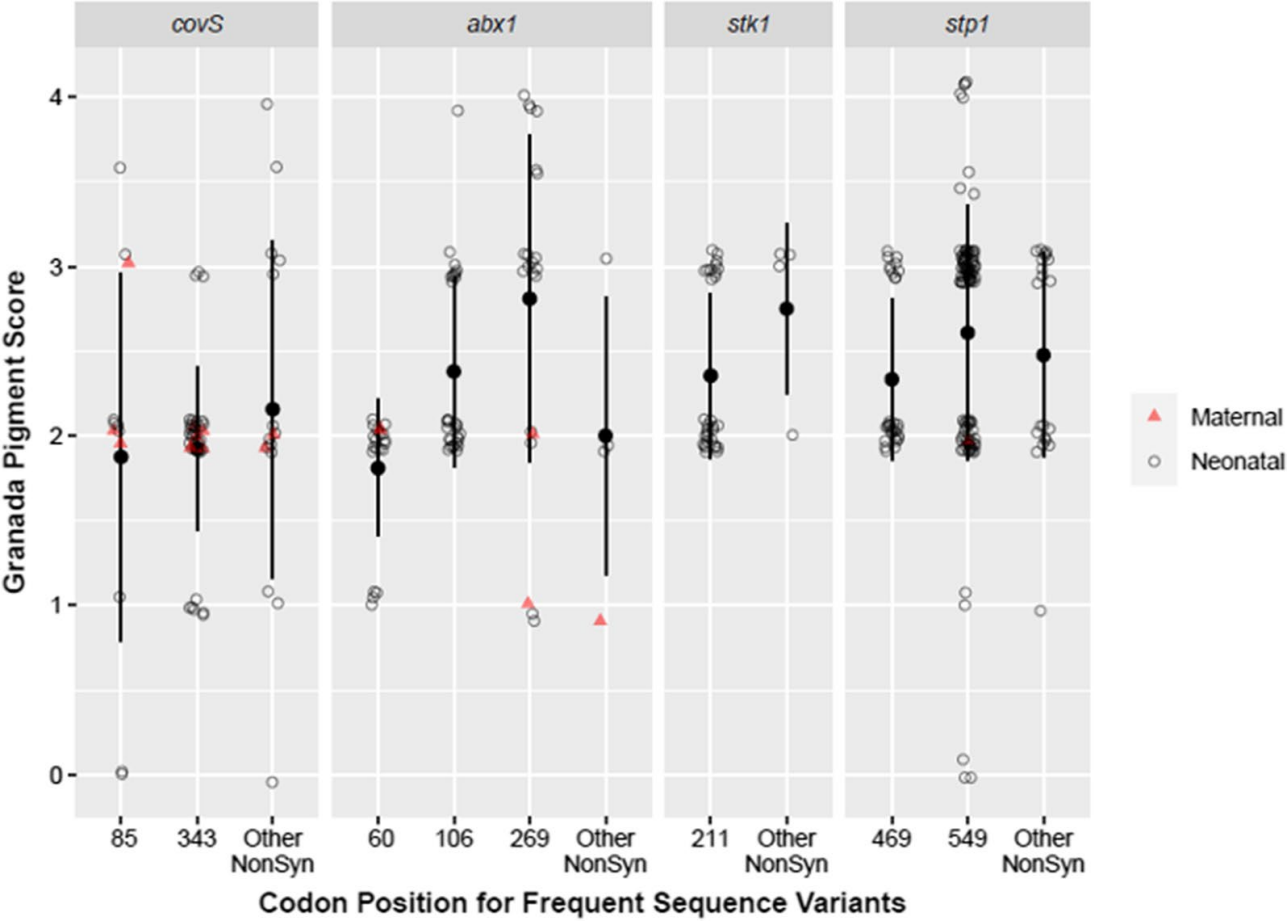
Scatter plot of Granada pigment score for maternal and neonatal isolates by amino acid position grouped by frequent non-synonymous mutations in *covR*, *covS*, *abx1*, and *stk1/stp1*. Isolates are represented with a dot for each non-synonymous mutation in the gene of interest and may be represented more than once

Correlation of GBS virulence factor expression with sequence variants and clinical outcome may lead to better prediction of populations at risk for perinatal GBS invasive disease. We sequenced *covR*, *covS*, *abx1*, *stk1* and *stp1*, genes which control GBS hemolysin (pigment) expression, and correlated sequence variants with Granada pigment score. All non-synonymous SNPs found in the coding regions of these genes represented either amino acid substitutions or a deletion of a base pair in *abx1* resulting in a frameshift mutation (Fig. 7).

One-third of GBS isolates had multiple non-synonymous mutations across these 5 genes (79/229, 34%). In the isolates with the highest pigment scores (Granada score ≥ 4), many sequence variants were observed resulting in amino acid substitutions that occurred in only one or two isolates: *covR* (g.A80V, g.A89D, g.V173I), *covS* (g.L47F, g.E237K, g.D275G, g.A285T, g.M322L), *abx1* (g.P16L), *stk1* (g.Q126H, g.E173D, g.G183S), and *stp1* (g.S124L, g.P138A, g.A216T, g.F439L, g.N497S, g.A534E). Overall, no pattern was observed for non-synonymous mutations within genes regulating *covR* and *covS* in isolates with the highest Granada pigment scores.

**Fig. 8:**
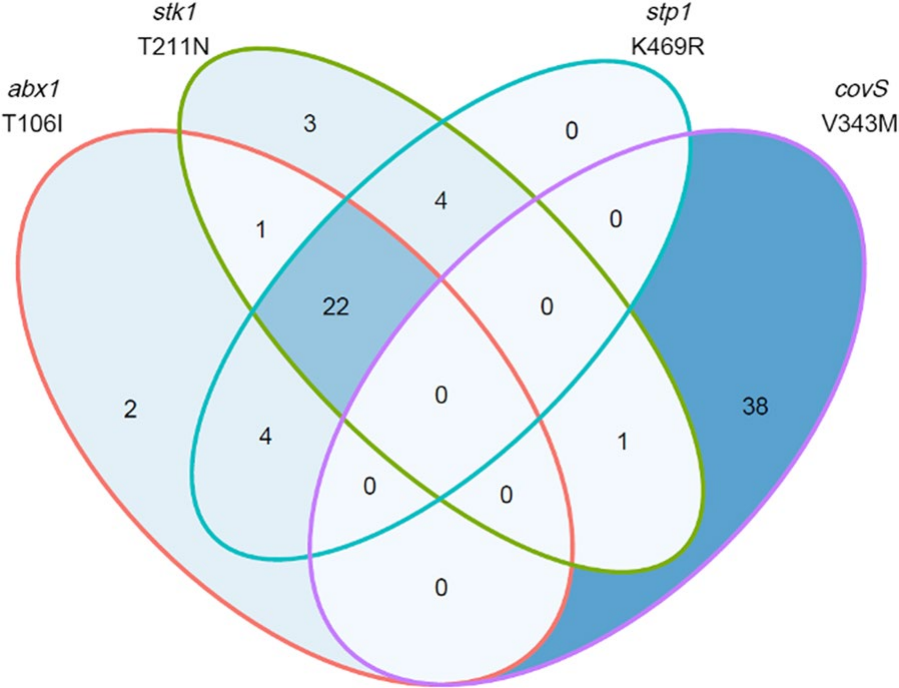
Venn diagram representing isolates with sequence variants in *abx1*, *stk1*, *stp1*, and *covS* that also had higher Granada pigment scores (2–4)*.* The red line encircles the *abx1* T106I variants, green line the *stk1* T211N variants, turquoise line the *stp1* K469R variants and the purple line the *covS* V343M variants

Some amino acid substitutions recurred in higher (Granada pigment score: 2–4) versus lower (Granada score < 2) pigmented isolates. In 37% (75/203) of higher pigmented isolates, at least one of four sequence variants were present that never or rarely occurred in the lower pigmented isolates: g.T106I (*abx1*), g.T211N (*stk1*), g.K469R (*stp1*) and g.V343M (*covS*). This skewed distribution of sequence variants occurring only in higher pigmented isolates was highly significant for each sequence variant independently. The g.T106I (*abx1*) and g.T211N (*stk1*) substitution, each occurred in 14% (29/203) of higher pigmented isolates and in no lower pigmented isolates (0/26, 0%; p < 0.001). Similarly, the sequence variant in *stp1* generating a g.K469R substitution was only found in higher pigmented isolates (30/203, 15%) and no lower pigmented isolates (0/26, 0%; p < 0.001). In addition, the g.V343M amino acid change in *covS* occurred in higher pigmented isolates significantly more often than in lower pigmented isolates (higher pigmented: 33/117, 28%; lower pigmented: 6/112, 5%; p < 0.001). Among the 203 higher-scoring pigmented GBS isolates, 22 (10.8%) had three of the four sequence variants and 10 (4.9%) had two of the four sequence variants (Fig. 8).

There was also a notable sequence variant in *abx1* of a deletion at nucleotide position 874 that generated a frame shift mutation, g.F60LfsX13, in the open reading frame. This variant occurred only in the lower pigmented isolates (Granada pigment score < 2) and never in higher pigmented isolates (21/112 vs 0/117, p < 0.001). Overall, there were significant differences in distribution of specific non-synonymous mutations in *covS*, *abx1*, *stk1* and *stp1*, which may be responsible for higher or lower β-hemolytic activity in the invasive GBS isolates in this Swedish cohort.

## Discussion

Knowledge of the molecular epidemiology of invasive and commensal GBS isolates is mainly restricted to the distribution of serotypes and multi-locus sequence types. However, GBS isolates are highly diverse and can regulate expression of adhesins and virulence factors in response to environmental stimuli, which are not represented by serotyping or multi-locus sequence typing. A deeper understanding of how GBS virulence factor expression is linked to invasion of immunocompromised hosts might enable a personalized medical approach to preventing perinatal GBS disease. This study provides the first analysis of virulence factor expression, phenotype, and genotype in a large collection of GBS isolates linked to clinical outcome. The main findings of our manuscript are: (1) a Granada or SBCB score ≥ 2 captured greater than 90% of all EOD isolates with excellent inter-rater reliability; (2) high hyaluronidase enzyme activity was observed in 16% of isolates and occurred in both pigmented and non-pigmented isolates; (3) a significantly higher proportion of maternal isolates had high hyaluronidase activity and lower Granada pigment scores compared to neonatal isolates, (4) hyaluronidase activity was negatively correlated with Granada pigment and SBCB scores, but not hemolysis score; (5) serotypes III and V were notable for their contrasting Granada pigmentation and hyaluronidase activity; and (6) common sequence variants in *stk1*, *stp1* and *covS* were expressed significantly more frequently in higher pigmented isolates suggesting that these variants may regulate CovR/S.

Targeting IAP to pregnant people that harbor highly virulent GBS isolates would reduce the global incidence of EOD but require a bacteriological assay to discriminate between GBS isolates with a high and low potential for invasion. We evaluated several assays to measure expression of the GBS hemolysin, which is regulated by the two-component system CovR/S that produces a hemolytic pigment; the pigment is the β-hemolysin and represents a key GBS virulence factor [18, 24, 40]. Although the hemolysis, Granada pigment and SBCB assays are all designed to detect expression of the beta-hemolysin, the Granada pigment and SBCB assays emerged as superior tests to capture the greatest proportion of invasive isolates in our cohort. It may be easier to distinguish differences in hemolysis on a colorimetric scale (Granada and SBCB pigment assays) and more difficult to accurately score slight differences in the zone of clearance around the colonies on blood agar (hemolysis assay). As a single test for expression of the GBS hemolysin, the Granada pigment assay captured the greatest number of invasive GBS isolates in our cohort. However, both the Granada pigment and SBCB assays captured > 90% of the invasive EOD isolates. The sensitivity and specificity of each of these microbial assays as predictors of adverse perinatal outcomes is unknown, may vary geographically and should be studied in populations with a high rate of perinatal invasive GBS disease.

Evaluation of hyaluronidase activity provides insight into a second GBS virulence factor that is regulated oppositely from pigment by CovR/S, in certain isolates. Increased hyaluronidase enzyme activity, especially in non-pigmented strains (e.g., GB37), suggests that immune suppression achieved by increased hyaluronidase activity promotes the virulence of these strains. Our results demonstrated that both pigmented and non-pigmented isolates can express high levels of hyaluronidase enzyme, suggesting that hyaluronidase is not always under CovR/S regulation. Recently, observations in a murine and nonhuman primate model linked the inoculation of GBS overexpressing hyaluronidase to rapid preterm labor and EOD, which demonstrates the potential for perinatal invasive disease with overexpression of this virulence factor [31, 33]. In our study, the elevated level of hyaluronidase in a case of GBS-associated stillbirth and maternal isolates is suggestive that hyaluronidase may be an important risk factor for invasive maternal and fetal infections. A follow-up test of low or non-pigmented isolates to determine hyaluronidase activity may help identify potentially hypervirulent isolates missed by screening of pigment alone. Interestingly, a higher activity of hyaluronidase was observed in isolates from pregnant and postpartum individuals than neonates; whether hyaluronidase expression is selectively advantageous in causing invasive disease in pregnant/postpartum individuals compared to neonates is unknown. The American Society for Microbiology guidelines highlight the importance of non-hemolytic (non-pigmented) GBS isolates in disease and recommend that further culture media and GBS isolation methods should detect both hemolytic and nonhemolytic strains.

Previous studies have shown that mutations in *covR/covS* alleviate repression of cyl operon resulting in hyperhemolytic GBS strains; further, overexpression of *abx1* or *stk1* or under-expression of *stp1* alleviates the CovR/S repression of the cyl operon. SNPs in *covS* may explain the virulence of some hyperhemolytic strains. We have previously found SNPs in *covS* (e.g., g.V343M) within invasive GBS isolates obtained from U.S. women in preterm labor [40]. Our findings in this Swedish cohort that the same *covS* substitution (g.V343M) commonly occurred in invasive isolates provides further evidence that this may be an important global sequence variant contributing to perinatal invasive disease. Hyperhemolytic GBS strains with phenotypes like c*ovR/covS* mutants have also been reported from infants and nonpregnant adults with severe invasive disease [22]. Furthermore, whole-genome comparison of 626 CC17 (Serotype III ST17) isolates revealed that frequent mutations in *covS* and *stk1* were a distinctive feature of disease-associated isolates [41]. In our cohort of Swedish invasive GBS isolates, we did not find premature stop codons or frameshift mutations that would have clearly disrupted activity of CovR. However, there were 4 sequence variants that recurred in 37% (75/203) of isolates with higher Granada pigment scores (2–4).

Study strengths include the considerable number of GBS invasive isolates collected over more than a decade with application of a variety of assays to characterize GBS virulence and genotype linked with clinical information. Evaluation of pigment (hemolysin) and hyaluronidase, two important GBS virulence factors that can be oppositely regulated by CovR/S, is a clear strength but also a limitation. Other GBS virulence factors may be involved in driving the invasive phenotypes of these isolates, which were not studied, including superoxide dismutase, cyclic di-AMP and CdnP, and D-alanylation of lipotechoic acid [20]. A second limitation is the lack of a geographically matched group of control isolates for the phenotypic assays, although we used a published Swedish GBS commensal group for comparison of CPS serotypes [36]. Note that universal screening for GBS is not performed in Sweden; further, the widespread use of IAP in the U.S. makes it impossible to determine whether a GBS isolate cultured near term could have resulted in an invasive infection, complicating the determination of invasive versus control isolates. Another potential limitation is that the phenotypic evaluations were all subjectively graded; however, the Kappa interrater coefficients were very high for these tests. It is also possible that due to differences in the components of culture media, there may be variability in the degree of expression and subsequent grading of Granada pigment. Finally, as we did not perform whole genome sequencing, we cannot estimate clonality in this study. Additional studies could build upon our results using invasive and control isolates from other geographic regions to understand the molecular epidemiology of GBS virulence factors, phenotype, and genotype. Additional studies could also assess other virulence factors that may play a role in GBS invasion, including sialylation of the capsular polysaccharide (responsible for immune evasion) or extracellular matrix proteins conferring host-cell adherence [20, 42, 43].

## Conclusions

Many countries do not administer intrapartum antibiotic prophylaxis for the prevention of EOD due to lower rates of GBS colonization and concern for increasing antibiotic resistance. There is enormous potential for a personalized medical approach to guide administration of intrapartum antibiotic prophylaxis based on expression of high-risk GBS virulence factors. Our findings suggest that testing for GBS pigmentation and hyaluronidase may, albeit imperfectly, identify pregnant people at risk for invasive disease. A Granada pigment or SBCB score ≥ 2 captured more than 90% of EOD isolates with high inter-rater reliability. Although testing for hyaluronidase expression is more complex, this may identify isolates that pose a particular risk for the mother or the fetus. The skewed distribution of several sequence variants in higher and lower pigmented isolates suggests a role in regulation of CovR/S. Additional studies are necessary to determine whether these virulence factors, phenotypes and sequence variants identified in a Swedish cohort are translatable to other geographic regions. However, the concept of using basic microbiological laboratory tests to determine the virulence potential of GBS isolates and guide the use of intrapartum antibiotics is an approach that needs to be explored.

## Data Availability

The data and materials presented in this study are available on request from Dr. Margret Gudjonsdottir (margret.johansson.gudjonsdottir@vgregion.se) and are subject to restrictions imposed by the European General Data Protection Regulation.

## Abbreviations

ABX1: Abi-domain protein-1
CAMP: Christie-Atkinson-Munch-Peterson
CPS: Capsular polysaccharide
CSF: Cerebrospinal fluid
EOD: Early-onset disease
GBS: Group B Streptococcus
IAP: Intrapartum antibiotics
LOD: Late-onset disease
PCR: Polymerase chain reaction
SBCB: Strep B Carrot Broth
SNP: Single nucleotide polymorphism
STK1: Serine/Threonine Kinase-1
STP1: Serine/Threonine Phosphatase-1
TLR: Toll-like receptor
TSA: Tryptic soy agar
TSB: Tryptic soy broth
U.S.: United States
